# Supplementary material for: Comparative multiomics analysis of cell physiological state after culture in a basket bioreactor
Source: Sci Rep. 2022 Nov 23;12:20161. doi: 10.1038/s41598-022-24687-4 (PMC9686226; doi:10.1038/s41598-022-24687-4)
Supplement: Supplementary file 1 — Supplementary Information 1. [file 41598_2022_24687_MOESM1_ESM.zip › raw data/Proteomic raw data/5.GOenrichment/EG.vs.CG/UP/EG.vs.CG.GO_Enrich.barplot.pdf]

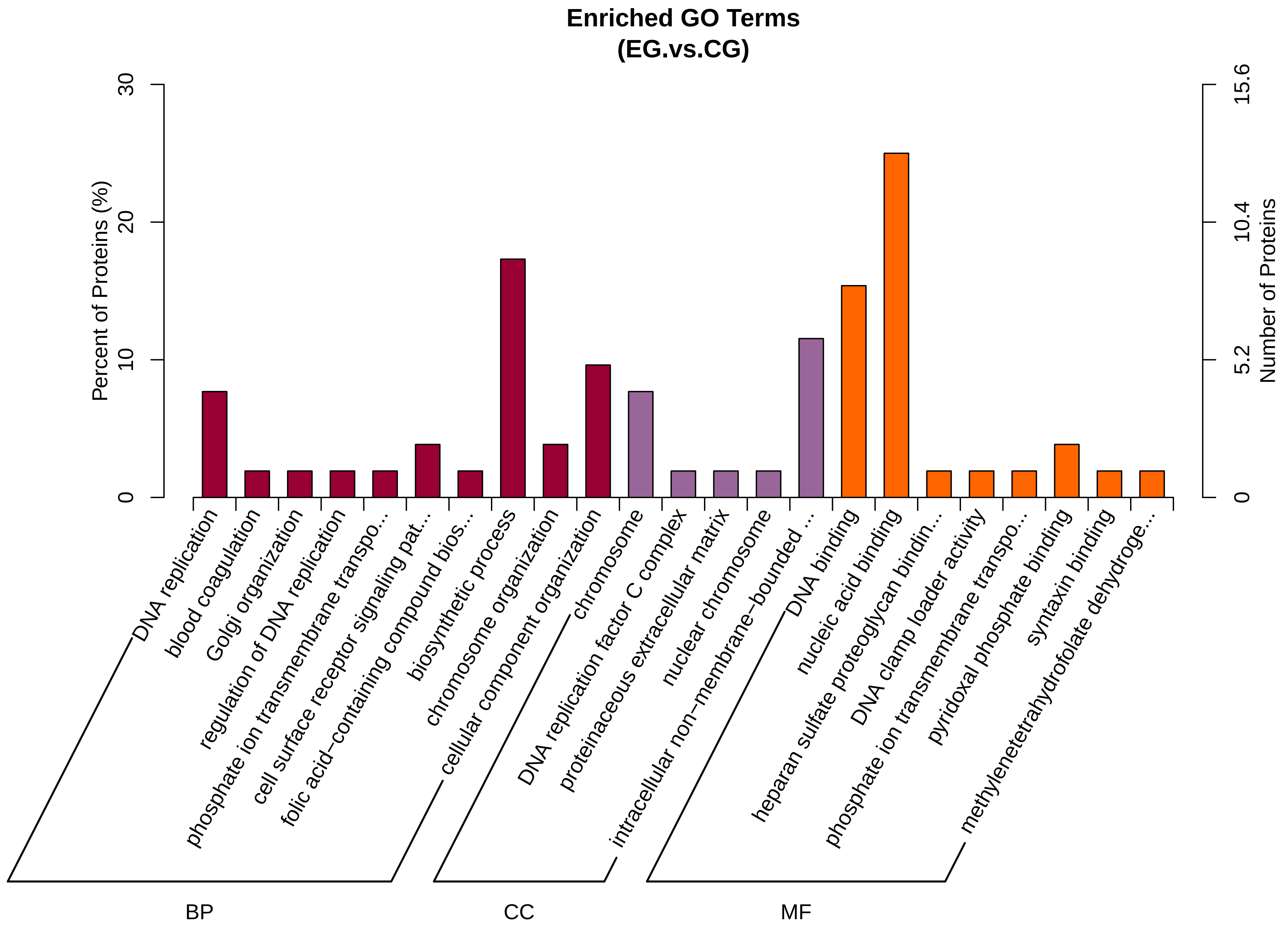
Enriched GO Terms
(EG.vs.CG)
Percent of Proteins (%)
Number of Proteins
0
10
20
30
0
5.2
10.4
15.6
DNA replication
blood coagulation
Golgi organization
regulation of DNA replication
phosphate ion transmembrane transpo...
cell surface receptor signaling pat...
folic acid–containing compound bios...
biosynthetic process
chromosome organization
cellular component organization
chromosome
DNA replication factor C complex
proteinaceous extracellular matrix
nuclear chromosome
intracellular non–membrane–bounded ...
DNA binding
nucleic acid binding
heparan sulfate proteoglycan bindin...
DNA clamp loader activity
phosphate ion transmembrane transpo...
pyridoxal phosphate binding
syntaxin binding
methylenetetrahydrofolate dehydroge...
BP
CC
MF
